# Low-intensity pulsed ultrasound enhances angiogenesis and ameliorates contractile dysfunction of pressure-overloaded heart in mice

**DOI:** 10.1371/journal.pone.0185555

**Published:** 2017-09-28

**Authors:** Tsuyoshi Ogata, Kenta Ito, Tomohiko Shindo, Kazuaki Hatanaka, Kumiko Eguchi, Ryo Kurosawa, Yuta Kagaya, Yuto Monma, Sadamitsu Ichijo, Hirofumi Taki, Hiroshi Kanai, Hiroaki Shimokawa

**Affiliations:** 1 Department of Cardiovascular Medicine, Tohoku University Graduate School of Medicine, Sendai, Japan; 2 Department of Electronic Engineering, Graduate School of Engineering, Tohoku University, Sendai, Japan; 3 Division of Biomedical Measurements and Diagnostics, Graduate School of Biomedical Engineering, Tohoku University, Sendai, Japan; Emory University, UNITED STATES

## Abstract

**Introduction:**

Chronic left ventricular (LV) pressure overload causes relative ischemia with resultant LV dysfunction. We have recently demonstrated that low-intensity pulsed ultrasound (LIPUS) improves myocardial ischemia in a pig model of chronic myocardial ischemia through enhanced myocardial angiogenesis. In the present study, we thus examined whether LIPUS also ameliorates contractile dysfunction in LV pressure-overloaded hearts.

**Methods and results:**

Chronic LV pressure overload was induced with transverse aortic constriction (TAC) in mice. LIPUS was applied to the whole heart three times in the first week after TAC and was repeated once a week for 7 weeks thereafter (n = 22). Animals in the control groups received the sham treatment without LIPUS (n = 23). At 8 weeks after TAC, LV fractional shortening was depressed in the TAC-Control group, which was significantly ameliorated in the TAC-LIPUS group (30.4±0.5 vs. 36.2±3.8%, P<0.05). Capillary density was higher and perivascular fibrosis was less in the LV in the TAC-LIPUS group than in the TAC-Control group. Myocardial relative ischemia evaluated with hypoxyprobe was noted in the TAC-Control group, which was significantly attenuated in the TAC-LIPUS group. In the TAC-LIPUS group, as compared with the control group, mRNA expressions of BNP and collagen III were significantly lower (both P<0.05) and protein expressions of VEGF and eNOS were significantly up-regulated associated with Akt activation (all P<0.05). No adverse effect related to the LIPUS therapy was noted.

**Conclusions:**

These results indicate that the LIPUS therapy ameliorates contractile dysfunction in chronically pressure-overloaded hearts through enhanced myocardial angiogenesis and attenuated perivascular fibrosis. Thus, the LIPUS therapy may be a promising, non-invasive treatment for cardiac dysfunction due to chronic pressure overload.

## Introduction

Heart failure (HF) is one of the major health problems with a prevalence of approximately 6 million in the United States (US) and more than 23 million worldwide [1–5]. In the US, 0.87 million people are newly diagnosed as having HF every year, and the number of HF patients is predicted to increase to 8 million by 2030 in the US [1]. The prevalence of hypertension and aortic stenosis has also been increasing along with population aging [6,7]. In patients with hypertension or aortic stenosis, the left ventricle (LV) is subjected to chronic pressure overload and the heart develops LV hypertrophy (LVH) as an adaptive response to increased workload, whereas sustained pressure overload causes maladaptive hypertrophy and decompensated HF [8]. Although it is not fully elucidated how pressure-overloaded hearts transit from compensated LVH to decompensated HF, a mismatch between the number of capillaries and the size of cardiomyocytes due to insufficient myocardial angiogenesis has been reported to be involved in the development of HF [9–12]. Indeed, compensated angiogenesis plays a crucial role in maintaining cardiac function in animal models of pressure-overload [10,12,13]. Vascular endothelial growth factor (VEGF) is crucial to maintain myocardial capillary density and reduced vascular bed is associated with the transition from compensated LVH to HF in response to pressure overload [14]. Thus, myocardial angiogenesis may be a promising therapeutic strategy in preventing the HF transition [15–18].

Ultrasound is an acoustic wave with frequency higher than 20 kHz, the upper audible limit of humans. Ultrasound is clinically used not only for diagnosis but also for therapeutic purposes, including tumor ablation, thrombolysis, bone regeneration, and drug delivery [19]. Low-intensity pulsed ultrasound (LIPUS) exerts anti- inflammatory effects [20], induces angiogenesis [21–24], and accelerates wound healing [25]. We have recently demonstrated that LIPUS induces angiogenesis and ameliorates LV dysfunction in a porcine model of chronic myocardial ischemia [26] and a mouse model of myocardial infarction (MI) [27]. However, it remains to be examined whether the LIPUS therapy is also effective to ameliorate LV dysfunction in non-ischemic heart disease, such as hypertensive heart disease and aortic valve stenosis. Thus, in the present study, we examined the effects of the LIPUS therapy on contractile dysfunction in LV pressure-overloaded hearts through therapeutic angiogenesis.

## Materials and methods

### Animal preparations

All animal experiments were performed conform the NIH guidelines (Guide for the care and use of laboratory animals) and were conducted in accordance with the protocols approved by the Institutional Committee for Use and Care of Laboratory Animals of Tohoku University (2013 Idou-547). Male C57BL/6 mice (9-week-old, 23–28 g in body weight) underwent transverse aortic constriction (TAC) to induce chronic LV pressure overload. They were separated every three or six individuals in the cages where was kept the temperature (22°C) and humidity (60%), and were given the food and water in the cages, and could access ad libitum. TAC procedures were partly modified and were performed as we have previously reported [28]. Briefly, the animals were anesthetized with 2.5–3.0% isoflurane and intubated and ventilated. After the left parasternal skin incision, the transverse aorta was exposed between the thymus gland, and 6–0 silk was placed under the transverse aorta. The transverse aorta was constricted with a 25-guage needle, which was removed immediately. Sham-operated animals underwent the same procedure without transverse aortic constriction. The chest wound was closed with a 6–0 silk suture. After the surgery, we observed the animals for 15 min on the warm plate and respiratory status. When the animal status was well controlled, it went back to the cages. If the respiratory status was unstable and the movement was poor after the surgery, the animal was euthanized at the end of the experiments by cervical dislocation after anesthetic inhalation of overdose with isoflurane. To distress the animal, intramuscular administration of ketamine (80 mg/kg) was used after TAC. The mortality of TAC surgery was approximately 20% and only the animals that survived for more than 24 hours after the surgery were used in the present study. Although we observed those animals every 3 hours after the surgery for 24 hours, some of the animals died without euthanasia within the first 24 hours probably due to sudden death caused by aortic rupture or lethal arrhythmia. Our institutional animal ethics committee reviewed and approved the mortality aspects of the protocol. One week after TAC, we measured flow velocity at the constricted aorta by Doppler flow measurement. The animals whose peak flow velocity at constriction was less than 4.0 m/sec were excluded from the present study. Animals were euthanized at the end of the experiments by cervical dislocation under anesthetic inhalation overdose with isoflurane.

### Blood pressure measurement

Blood pressure was measured at baseline and 8 weeks after TAC with the tail-cuff system (Muromachi Kikai Co, Ltd, MK-2000ST NP-NIBP Monitor, Tokyo, Japan) without anesthesia. We also measured blood pressure at 1 week after TAC or sham surgery for 60 min at an interval of 5 min with the tail-cuff system under anesthesia with inhaled isoflurane (1.2–1.5%).

### Transthoracic echocardiography

Transthoracic echocardiography was performed before and 1, 2, 4, 6 and 8 weeks after TAC to evaluate contractile function under light anesthesia with inhaled isoflurane (0.4–1.5%). The heart rate was kept more than 500 beats/minute [29]. Two-dimensional M-mode echocardiography was performed with Vevo 2100 (Visual Sonics, Ontario, Canada). Cardiac dimensions, such as LV systolic and diastolic dimension (LVDs, LVDd) and anterior and posterior wall thickness (AWT, PWT), was measured. Cardiac contractile function represented by LV fractional shortening (LVFS) was calculated as [(LVDd-LVDs)/LVDd] ×100. LV ejection fraction (LVEF) was calculated as 100×[(LVEDV-LVESV)/LVEDV], where LV end-diastolic (systolic) Volume (LVED(S)V) was calculated as [(7.0/(2.4+LVDd (s)] ×LVDd(s)^3^.

### LIPUS therapy

**Fig 1:**
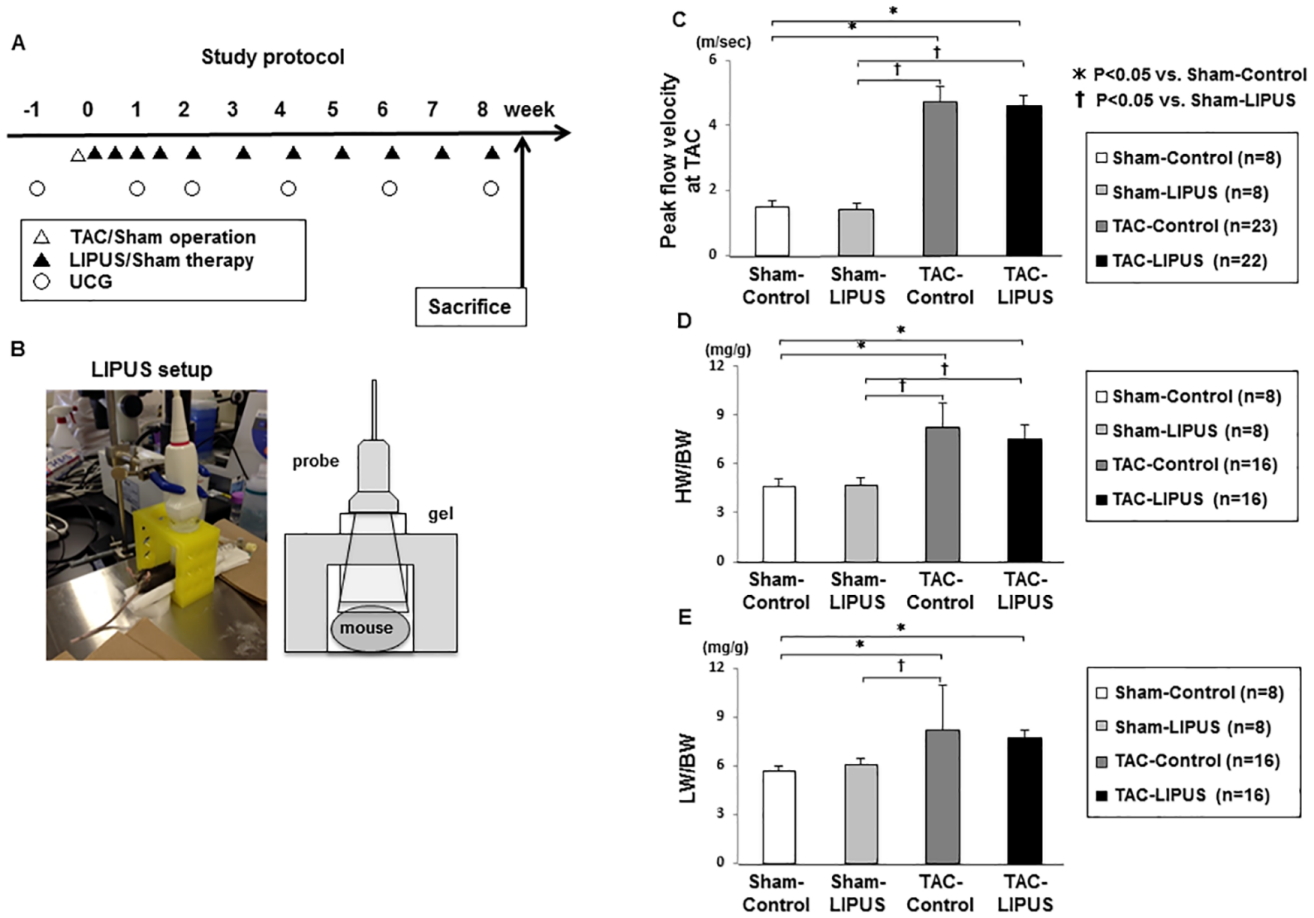
Study protocol. (A) Study protocol. LIPUS was applied to the whole heart three times in the first week after TAC and was thereafter repeated once a week for 7 weeks in the LIPUS group, while the control group underwent the same procedures but without the LIPUS therapy. (B) Study setup. (C) Peak flow velocity at TAC. (D) Heart weight/body weight (HW/BW). (E) Lung weight /body weight (LW/BW). Results are expressed as mean±SD.

For the LIPUS therapy, we used a diagnostic ultrasound device (Prosound α10; HITACHI Aloka Medical, Ltd., Mitaka, Japan) whose irradiation conditions could be modified by software modification and a sector-type probe that is commercially available as a diagnostic device for humans. Based on our previous studies [26,27], we performed the LIPUS therapy under the following conditions; frequency 1.875 MHz, pulse repetition frequency 4.90 kHz, number of cycles 32, voltage applied to each transducer element, 17.67 volts (V), and Ispta (spatial peak temporal average intensity) 117–162 mW/cm^2^. The power of LIPUS was 0.25 W/cm^2^, the beams were irradiated from the sector-shaped probe and were focused at 6 cm depth. The voltage applied to each transducer element was controlled to keep estimated Ispta of LIPUS below the upper limit of acoustic output standards (<720 mW/cm^2^) for diagnostic ultrasound devices (US Food and Drug Administration’s Track 3 Limits) and to prevent the ultrasound probe from temperature rise. LIPUS was applied to mice through an agar phantom gel (Fig 1B). The attenuation coefficient of the phantom gel was almost comparable to that of living cells (e.g. muscles, fat, and blood). LIPUS was applied to the heart by 2-dimensional scan at 3 different short axis levels (basal LV, papillary muscles, and apex for 20 min each) in a day under anesthesia with inhaled isoflurane (1.2–1.5%) [27]. Mice in the LIPUS group were subjected to the LIPUS therapy 3 times in the first week (1, 3 and 5 days after TAC) and subsequently repeated once a week for 7 weeks. Animals in the control group underwent the same procedures including anesthesia but without the LIPUS therapy.

### Histological analysis

Excised hearts were fixed with 4% formalin for histological and immunohistochemical examinations. The tissue specimens were embedded in paraffin and sliced at 3-μm in thickness. The sections were stained by hematoxylin-eosin (HE) and Masson-trichrome (MT); the latter was used to assess LV myocardial fibrosis. The extent of perivascular fibrosis was quantified as previously described [30]. Briefly, following MT staining, images of myocardial coronary arteries ranging from 50 to 200 μm in diameter were obtained. Perivascular fibrosis was identified by blue staining, and the smooth muscle was identified by red staining. The area of perivascular fibrosis was calculated by subtracting the area of the vessel (lumen + smooth muscle) from the area of the fibrosis + the vessel, using the Image J Software (NIH, USA). CD31 and Mac-3 immunostaining were used to assess myocardial capillary density and macrophage infiltration, respectively. The hypoxic regions in the heart were evaluated by hypoxyprobe-1 kit (HP1-100Kit, Hypoxyprobe Inc., MA, USA), according to the manufacturer’s instructions [28]. Pimonidazole was injected intraperitoneally at a dose of 60 mg/kg body weight, 30–90 min prior to sample harvest [28]. Semi-quantitative analysis regarding the extent of hypoxyprobe was evaluated for each image by using the following scale; 0 = none, 1 = slight, 2 = moderate, and 3 = high, as previously described [31–34].

### RNA isolation and real time RT-PCR

Isolation of total RNA from mouse heart tissues was performed using the RNeasy Plus Mini Kit (Qiagen, Hilden, Germany) according to the manufacturer’s protocol. Total RNA was converted to cDNA using PrimeScript RT Master Mix (Takara Bio Inc., Kusatsu, Japan). Primers for murine *Nppa* (Nppa, Primer Set ID, MA102516), *Nppb* (Nppb, Primer Set ID, MA103081), *Col1a1* (Collagen Iα1, Primer Set ID, MA107374), *Col3a1* (Collagen IIIα1, Primer Set ID, MA131765), *Myh6* (αMHC, Primer Set ID, MA103418), *Myh7* (βMHC, Primer Set ID, MA173907), and *Gapdh* (GAPDH, Primer Set ID, MA050371) were purchased from Takara Bio Inc. After reverse transcription, real-time RT-PCR was performed by CFX96^™^ Real-time system C1000^™^ Thermal Cycler (Bio-Rad, CA, USA), using SYBR Premix Ex Taq II (Takara Bio Inc.) for SYBR probes. We assessed the relative expression of targeted mRNA to glycerol-3-phosphate dehydrogenase (GAPDH).

### Western blot analysis

An equal amount of protein was loaded on SDS-PAGE gel and transferred to PVDF membranes (GE Healthcare, Buckinghamshire, UK), and blocked for 1 hour at room temperature in 5% BSA or skim milk in Tris-buffered saline with Tween 20 (TBST). The primary antibodies were used as follows; VEGF (1000:1, Santa Cruz, sc-152), phosphorylated-eNOS (Ser1177) (1000:1, BD, #612393), total-eNOS (1000:1, Enzo, ADI-905-386), phosphorylated-ERK1/2 (Thr202/Tyr204) (1000:1, Cell Signaling, #9101), total-ERK1/2(1000:1, Cell Signaling, #9102), phosphorylated-protein kinase B (Akt) (Ser473) (1000:1, Cell Signaling, #4060), total-Akt (1000:1, Cell Signaling, #9272), CD31 (1000:1, Abcam, ab32457), HGF (500:1, abcam, ab83760), basic FGF (500:1, Santa Cruz, sc-79) and α-tubulin (1000:1, Sigma, T9026). The regions containing protein were visualized by the enhanced chemiluminescence system (ECL Prime Western Blotting Detection Regent, GE Healthcare, Buckinghamshire, UK). Densitometric analysis was performed by the Image J Software (NIH, USA).

### Measurement of cytokines/chemokines and growth factors by the Bio-Plex system

Cytokines/chemokines and growth factors were evaluated by the Bio-Plex System, according to the manufacturer’s instructions (Bio-Rad, Tokyo, Japan). Mouse cytokines/chemokines and growth factors in the serum were measured with commercially available kits (Bio-Rad, 9-Plex, and 23-Plex) in mice with or without the LIPUS therapy after TAC.

### Statistical analysis

Statistical analysis was performed with Excel (Microsoft, WA, USA) and JMP (SAS Institute Inc., NC, USA). Results are shown as mean ± standard deviation (SD) for all experiments. Comparisons of mean parameters among multiple groups were performed by one-way ANOVA, followed by Turkey’s HSD multiple comparison test. Echocardiography parameters were compared at 8 weeks after TAC. For all experiments, P values < 0.05 were considered to be statistically significant.

## Results

### Effects of the LIPUS therapy on TAC-induced pressure-overloaded heart

The present protocol includes the LIPUS therapy three times a week after TAC, followed by once-a-week therapy for 7 weeks (Fig 1). Before finalizing this protocol, we also performed a preliminary study, in which the LIPUS was performed only three times a week after TAC with no subsequent therapy (S1 Fig). Since the therapeutic effects of LIPUS on cardiac function were not sustained in this preliminary protocol (S1 and S2 Figs), we added once-a-week LIPUS therapy for 7 weeks after TAC (Fig 1A).

Mice were subjected to transverse aortic constriction (TAC) or sham surgery and were then followed up for 8 weeks (Fig 1A and 1B) [28,29]. One week after the surgery, the peak flow velocity at the constriction in TAC-operated groups was significantly faster than that at the aorta in sham-operated groups, however there was no difference in the peak flow velocity between the TAC-operated groups (Fig 1C). There was no difference in survival rate during the follow-up for 8 weeks between TAC-Control group and TAC-LIPUS group (82% vs. 77%, P = 0.74). There was no difference in heart or lung weight between the TAC-operated groups, although the weights were significantly higher in the TAC-operated group than in the Sham-Control group (Figs 1D, 1E, S3A and S3B). Also, there was no difference in systolic blood pressure between the TAC-operated groups (114±10 vs. 117±11 mmHg at 8 weeks). There was no difference in systolic blood pressure during the experiment between the groups with or without the LIPUS therapy (S3C Fig).

**Fig 2:**
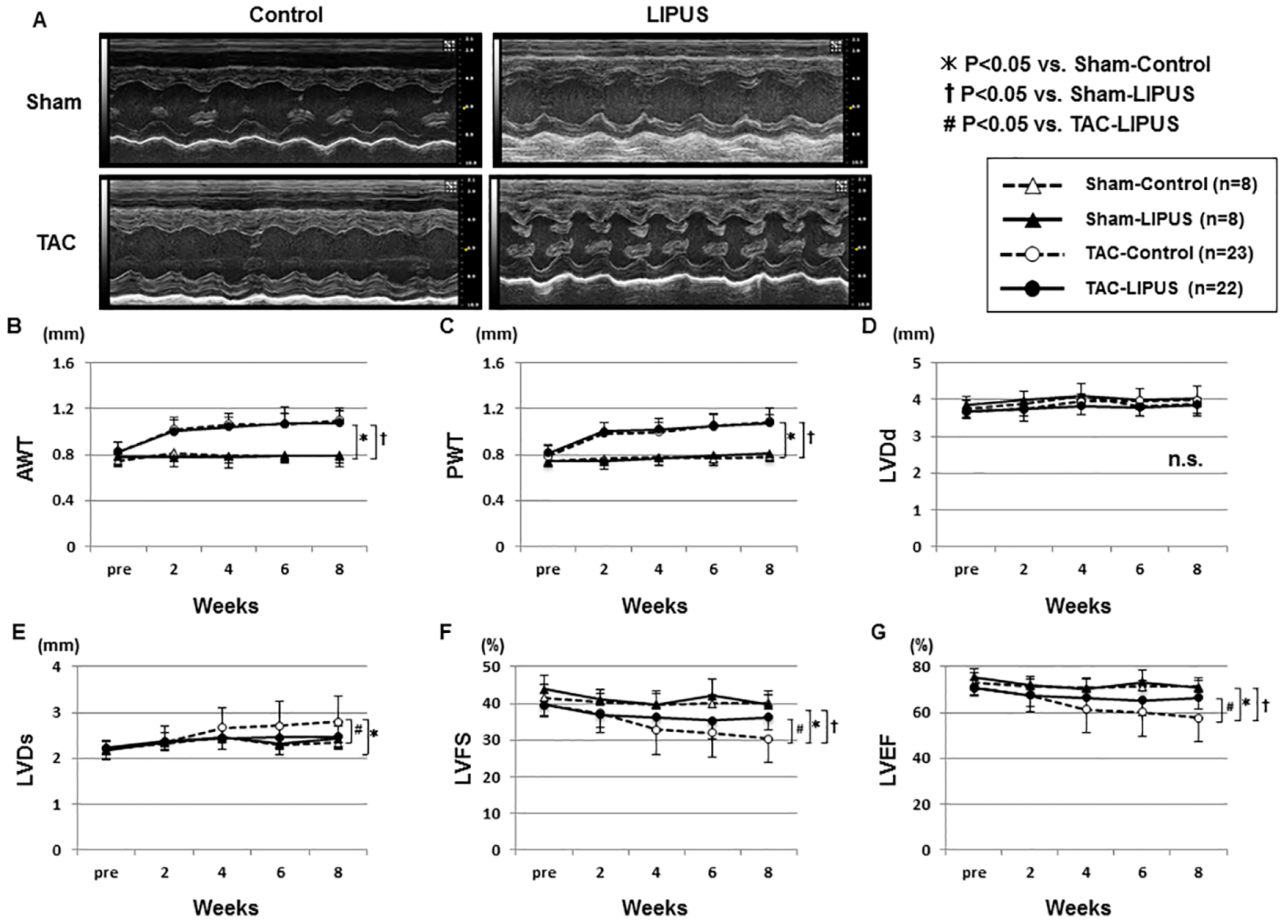
The LIPUS therapy ameliorates cardiac contractile dysfunction. (A) Representative echocardiographic images at 8 weeks after TAC. (B~G) Graphs showing the time course of anterior wall thickness (AWT) and posterior wall thickness (PWT) of the LV, LV dimension at end-diastole (LVDd), LVD at end-systole (LVDs), LV fractional shortening (LVFS), and LV ejection fraction (LVEF). Results are expressed as mean±SD. n.s., not statistically significant. Statistical analysis was performed at 8 weeks after TAC.

Echocardiographic study showed that LV wall thickness was significantly higher in the TAC-operated groups than in the Sham-operated groups, although there was no difference in LV wall thickness between the TAC-operated groups (Fig 2B and 2C). Also, there was no difference in LV end-diastolic dimension (LVDd) between the groups (Fig 2D), whereas LV end-systolic dimension (LVDs) was significantly smaller in the TAC-LIPUS group than in the TAC-Control group (LVDs at 8 weeks, 2.5±0.2 vs. 2.8±0.6 mm, P<0.05) (Fig 2E). In the TAC-LIPUS and TAC-Control groups, there was a positive correlation between LVDd and LVDs in each animal (S4 Fig). LV contractile function, when evaluated by LV fraction shortening (LVFS) and LV ejection fraction (LVEF), was progressively depressed in the TAC-Control group, which was significantly ameliorated in the TAC-LIPUS group (LVFS at 8 weeks, 30.3±6.5 vs. 36.2±3.6%; LVEF at 8 weeks, 57.7±10.3 vs. 66.4±4.7%, both P<0.05) (Fig 2F and 2G). No complications, such as arrhythmias or skin burns, were noted during the experimental period.

### Effects of the LIPUS therapy on myocardial ischemia and fibrosis in the pressure-overloaded heart

**Fig 3:**
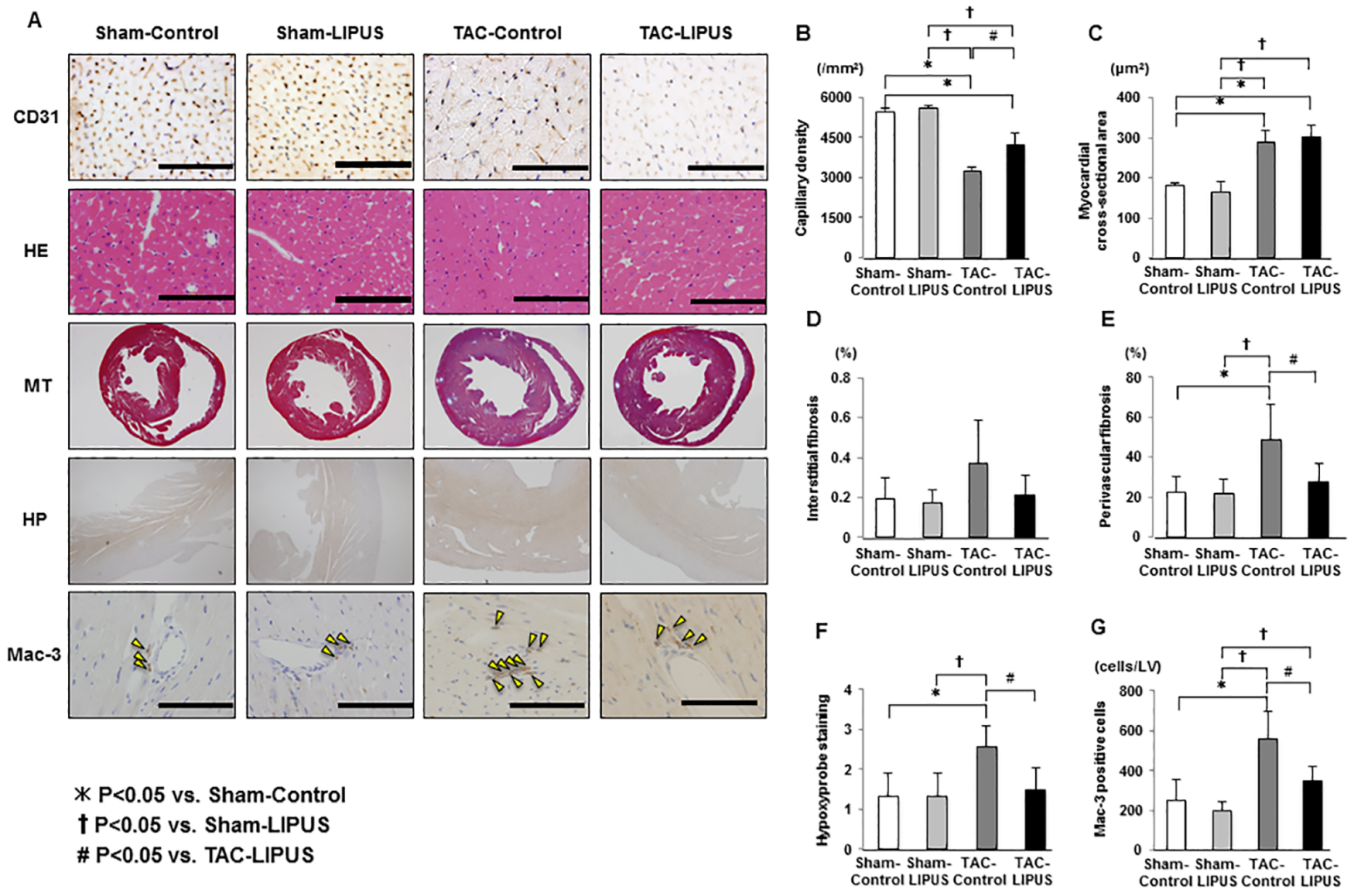
The LIPUS therapy attenuates perivascular fibrosis, myocardial ischemia and macrophage infiltration. (A) Representative histological images at 8 weeks after TAC. HE, hematoxylin-eosin staining; MT, Masson-trichrome staining; HP, Hypoxyprobe staining. (B~E) Graphs showing capillary density, myocardial cross-sectional area, interstitial fibrosis, and perivascular fibrosis(n = 3, 3, 6, 6 for each group). (F) Semi-quantitative analysis of hypoxyprobe staining(n = 3, 3, 7, 6 for each group). (G) Graph showing the number of Mac-3 positive cells(n = 3, 3, 5, 5 for each group). Results are expressed as mean±SD. Scale bars indicate 100 μm.

We have previously reported that the LIPUS therapy induces angiogenesis in a porcine model of chronic myocardial ischemia [26] and a mouse model of MI [27]. In the present study, capillary density in the LV was significantly higher in the TAC-LIPUS group than in the TAC-Control group (4229±455 vs. 3243±143 /mm^2^, P<0.005) (Fig 3A and 3B). There was no difference in myocardial cross-sectional area between the two groups (Fig 3C). Although there was no difference in myocardial interstitial fibrosis between the two groups, perivascular fibrosis was significantly less in the TAC-LIPUS group than in the TAC-Control group (27.8±9.3 vs. 48.9±17.7%, P<0.05) (Fig 3D and 3E), which was associated with a reduction in macrophage infiltration (Fig 3G). There results suggest that the LIPUS therapy preserves LV contractile function after TAC associated with enhanced myocardial angiogenesis and attenuated perivascular fibrosis in the LV.

Since relative ischemia has been reported to be involved in the transition from compensated hypertrophy to decompensated HF [10], we evaluated the effects of the LIPUS therapy on the extent of myocardial ischemia in pressure-overloaded hearts with hypoxyprobe [28]. Myocardial ischemia evaluated with hypoxyprobe was noted in the TAC-Control group, which was significantly attenuated in the TAC-LIPUS group (Fig 3F).

### Effects of the LIPUS therapy on angiogenic signaling pathways in vivo

**Fig 4:**
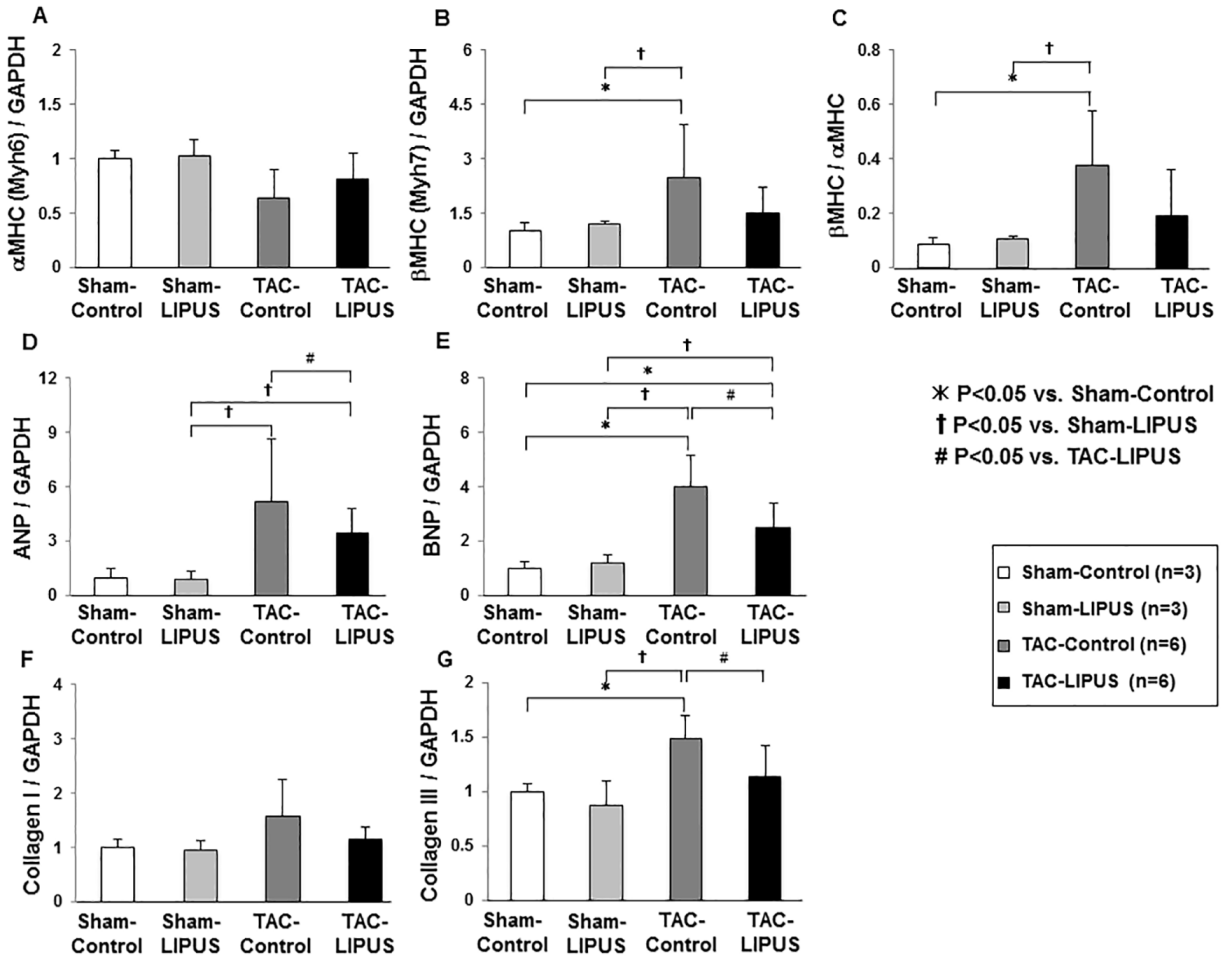
The LIPUS therapy down-regulates mRNA expression of BNP and collagen III. mRNA expression of α-MHC, β-MHC, atrial natriuretic peptide (ANP), brain natriuretic peptide (BNP), collagen I, and collagen III in the chronic phase (n = 3, 3, 6, 6 for each group). Results are expressed as mean±SD.

**Fig 5:**
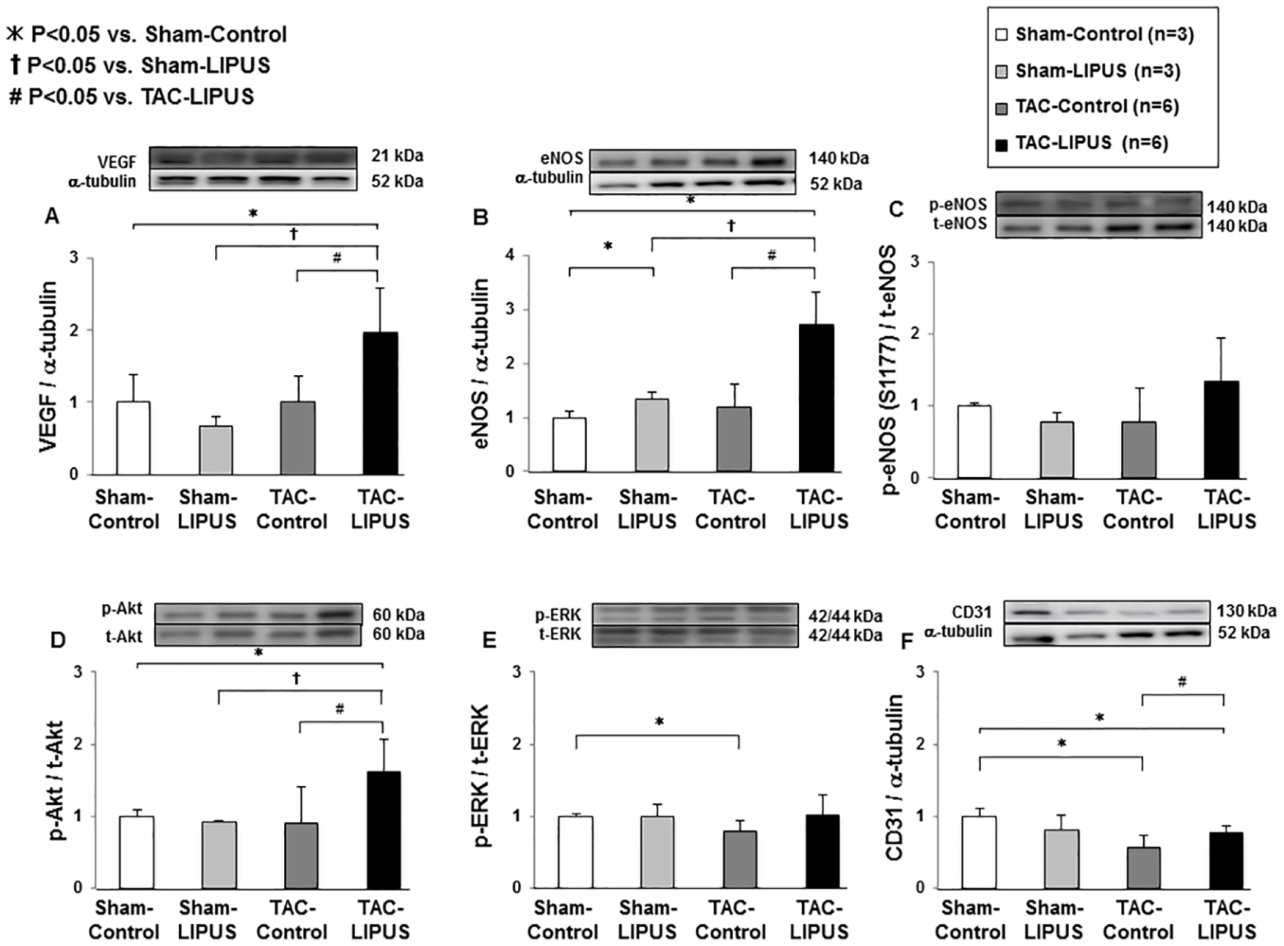
The LIPUS therapy activates the angiogenic signaling pathways. Protein levels of VEGF and eNOS, phosphorylation state of eNOS (Ser1177), Akt and ERK1/2, and protein levels of CD31 (n = 3, 3, 6, 6 for each group). Results are expressed as mean±SD.

We have previously reported that β_1_-integrin and caveolin-1 are the key molecules in the LIPUS-induced therapeutic angiogenesis, which play crucial roles in mechano-transduction and subsequent activation of angiogenic signal pathways such as VEGF and endothelial NO synthase (eNOS) [27]. We thus examined the effects of the LIPUS therapy on those signaling pathways in the pressure-overloaded hearts in vivo. Although the extent of mRNA expression of α-MHC and β-MHC was similar in the two groups, that of BNP and collagen III was lower in the TAC-LIPUS group than in the TAC-Control group (Fig 4). In the acute phase after TAC, the LIPUS therapy up-regulated the protein levels of VEGF, eNOS, and CD31, and enhanced the phosphorylation of Akt, but not that of ERK1/2 (Figs 5 and S5).

Although there were no differences in the circulating levels of VEGF and other angiogenic factors between the TAC-LIPUS and TAC-Control groups (S6 Fig), the protein expression of HGF was significantly up-regulated in the acute phase and that of bFGF in the chronic phase in the LV (S7 Fig). These results suggest that the LIPUS therapy affects the expression of multiple growth factors in pressure-overloaded hearts.

## Discussion

**Fig 6:**
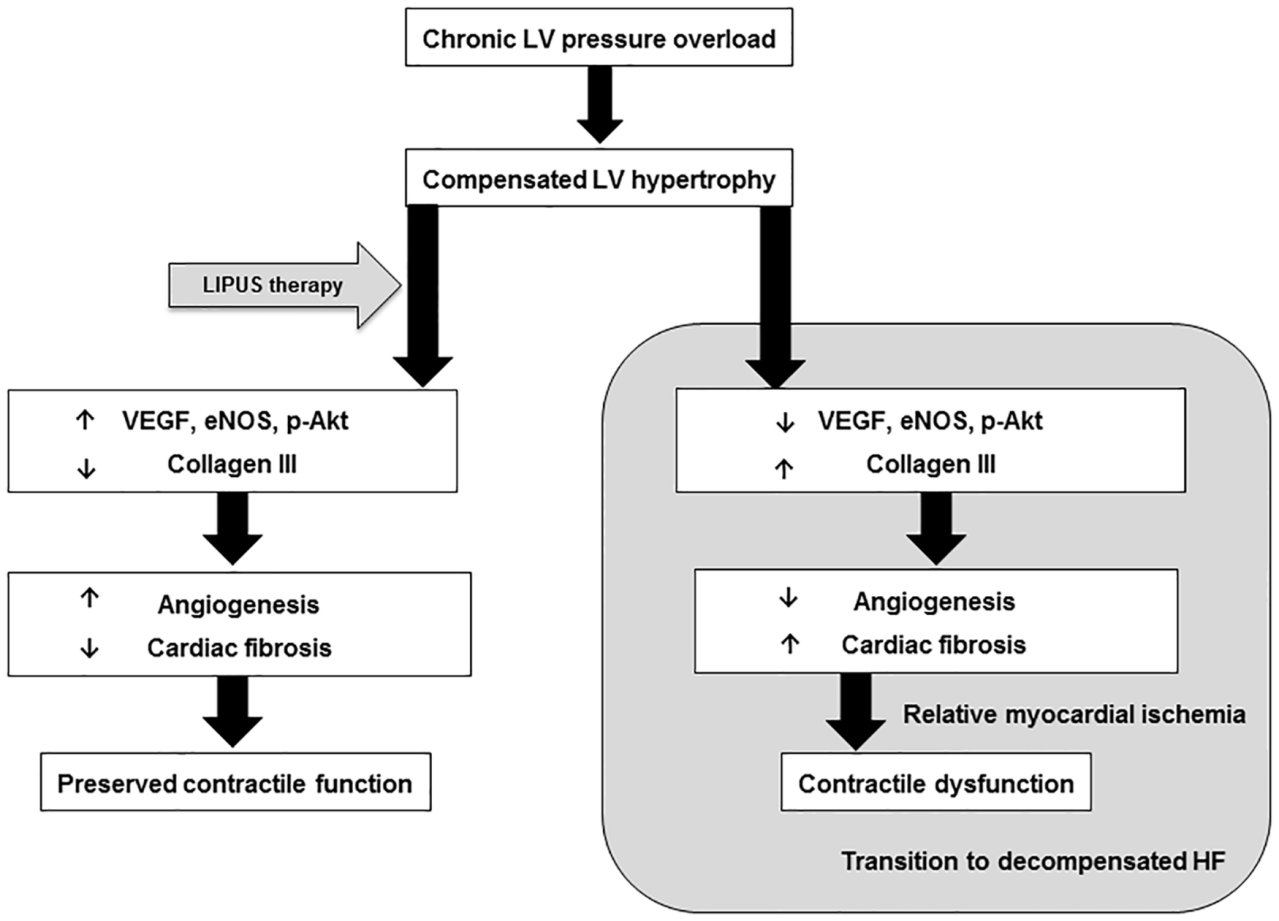
Intracellular signaling pathways for the beneficial effects of the LIPUS. The LIPUS therapy ameliorates cardiac contractile dysfunction by enhancing angiogenesis through up-regulation of VEGF, eNOS and p-Akt and attenuating perivascular fibrosis through down-regulation of collagen III in the LV, suppressing the transition from compensated LVH to decompensated HF.

In the present study, we were able to demonstrate that the LIPUS therapy ameliorates LV contractile dysfunction in TAC-induced pressure-overloaded hearts in mice in vivo, where enhanced myocardial angiogenesis and attenuated perivascular fibrosis in the LV may play a pivotal role in the beneficial effects of the LIPUS (Fig 6). To the best of our knowledge, this is the first report demonstrating that the LIPUS therapy is effective to maintain contractile function of chronically LV pressure-overloaded hearts. These results imply that the LIPUS might be effective to ameliorate LV dysfunction in patients with non-ischemic heart disease, such as hypertensive heart disease and aortic valve stenosis, in addition to patients with IHD.

### Beneficial effects of the LIPUS therapy on LV contractile dysfunction in TAC mice

In the present study, the LIPUS therapy ameliorated LV contractile dysfunction caused by chronic pressure overload, associated with enhanced myocardial angiogenesis and suppressed perivascular fibrosis in the LV. These beneficial effects were observed when the LIPUS was applied to the hearts three times in the first week after TAC and was repeated once a week for 7 weeks thereafter (Figs 1 and 2). However, when the LIPUS was applied only three times in the first week after TAC without subsequent therapies, these LIPUS-induced beneficial effects were not sustained (S1 and S2 Figs). These results suggest that the repetitive LIPUS therapies are necessary for sustained beneficial effects in chronically pressure-overloaded hearts.

### Possible mechanisms for the beneficial effects of LIPUS

We have previously demonstrated that the LIPUS therapy enhances the expression of VEGF and phosphorylation of eNOS, induces angiogenesis, and ameliorates LV dysfunction in a porcine model of chronic myocardial ischemia [26]. In the present study, capillary density in the LV was significantly higher in the LIPUS group than in the control group while there was no difference in heart weight or myocardial cross sectional area between the two groups. Also, the LIPUS therapy significantly attenuated macrophage infiltration and myocardial perivascular fibrosis. Furthermore, hypoxyprobe staining revealed the presence of relative myocardial ischemia in the control group, which was also ameliorated in the LIPUS group. Relative myocardial ischemia, a mismatch between the number of capillaries and the size of cardiomyocytes due to impaired cardiac angiogenesis, has been reported to be involved in the progression from LV compensated hypertrophy to decompensated HF [8–11]. Also, sustained compensated angiogenesis plays a crucial role in maintaining cardiac function in chronically pressure-overloaded hearts [10,12,13]. These results suggest that the LIPUS therapy ameliorates myocardial ischemia through sustained angiogenic effects and attenuated perivascular fibrosis, although it is unclear whether attenuation of myocardial interstitial fibrosis, but not that of interstitial fibrosis, contributes to preserved the LV contractile function.

We have previously reported that β_1_-integrin and caveolin-1 are the key molecules in the LIPUS-induced therapeutic angiogenesis [27]. To study the contribution of caveolin-1 to the beneficial effects of LIPUS on LV pressure overloaded hearts in vivo, we examined the effects of the LIPUS therapy in caveolin-1-knockout (Cav-1-KO) mice (S1 Text, S8 and S9 Figs). In the present study, we demonstrated that the beneficial effects of the LIPUS therapy on contractile dysfunction in LV pressure- overloaded hearts were blunted in Cav-1-KO mice, suggesting an important role of caveolin-1 in the LIPUS-induced therapeutic effects in pressure-overloaded hearts. Since β1-integrin- knockout mice are embryonic lethal [27], we did not examine the role of β1-integrin.

### Intracellular signaling pathways for the beneficial effects of the LIPUS therapy

VEGF signaling has been reported to play a crucial role in promoting angiogenesis and restoring blood supply to ischemic tissues in various pathological conditions, including LV pressure overload-induced HF [13,35,36]. Insufficient angiogenic response to myocardial hypoxia leads to cardiac dysfunction [34], while administration of a VEGF trap reagent delays transition from compensated hypertrophy to decompensated HF in pressure-overloaded hearts [14]. Also, Akt, a serine/threonine protein kinase, regulates cardiac growth, myocardial angiogenesis, glucose metabolism, and cell death in cardiomyocytes [37]. In cardiomyocytes, short-term Akt activation improves contractile function in pressure overload-induced HF [38]. The Akt signaling pathway is essential in VEGF-mediated post-neonatal angiogenesis [39]. We have previously demonstrated that the LIPUS therapy up-regulates mRNA expression of VEGF in human umbilical vein endothelial cells (HUVECs) and in human cardiac myocytes (HCMs), but not in human cardiac fibroblasts (HCFs) in vitro [27]. In the present study, the LIPUS therapy significantly up-regulated protein levels of VEGF and p-Akt. Thus, we consider that endothelial cells and cardiac myocytes play important roles in the beneficial effects of LIPUS. Furthermore, Akt phosphorylation was enhanced in the acute phase after TAC, implying that a reduced apoptotic cell death might contribute to the LIPUS-induced angiogenesis in addition to increased proliferation of endothelial cells. However, it is unclear whether interactions among different kinds of cells cause additional effects. This point remains to be examined in future studies.

eNOS is known as an important regulator of cardiac function as LVH and contractile function in response to LV pressure overload are deteriorated in eNOS^-/-^ mice compared with wild-type mice [40,41]. In the present study, eNOS expression in the LV was also enhanced by the LIPUS therapy, suggesting that the LIPUS therapy preserves LV function through the VEGF/Akt and eNOS pathways.

Although collagen type I is ubiquitously expressed, collagen type III is expressed in a relatively tissue-specific manner, which is highly expressed in the skin, lungs, and blood vessels [42]. In the present study, in the LIPUS group as compared with the control group, the expression of collagen type III was attenuated in the chronic phase. Furthermore, macrophage infiltration and myocardial perivascular fibrosis were significantly less in the LIPUS group than in the control group. These results suggest that the LIPUS therapy attenuates perivascular fibrosis by suppressing inflammatory responses and the expression of collagen type III, all of which effects may contribute, at least in part, to the beneficial effects of LIPUS. Taken together, both enhanced angiogenesis through up-regulation of VEGF, eNOS and p-Akt and attenuated perivascular fibrosis through down-regulation of collagen III may ameliorate relative myocardial ischemia and contractile dysfunction.

### Clinical implications

Hypertension is one of the major risk factors for HF. Although we have previously demonstrated that the LIPUS therapy is effective to ameliorate HF in a pig model of chronic myocardial ischemia [26] and a mouse model of MI [27], we confirmed that the LIPUS therapy is also effective in a mouse model of chronic LV pressure-overload in the present study. These results suggest that the LIPUS therapy may be effective for the treatment of patients with hypertensive heart disease or aortic stenosis.

In the present study, no complications were noted as the intensity of ultrasound used is below the upper limit of the regulation for diagnostic devices. Due to its non-invasive nature, the LIPUS therapy is feasible even for elderly patients or complicated patients. We are now conducting a clinical trial of the LIPUS therapy in patients with refractory angina pectoris. Hypertensive heart disease and aortic stenosis would be other possible indications for the LIPUS therapy.

### Study limitations

Several limitations should be mentioned for the present study. First, although we have demonstrated the beneficial effects of the LIPUS therapy in HF models, including chronic myocardial ischemia in pigs [26], AMI in mice [27], and as shown in the present study, in TAC in mice, it is unclear whether the LIPUS therapy is also beneficial in other HF models, such as doxorubicin-induced cardiomyopathy model or Dahl salt-sensitive rat model. Second, when the transverse aorta was constricted with a 27-guage needle to induce LV pressure overload in the preliminary study, the mice progressively developed heart failure (HF) and most of them died during anesthesia for LIPUS therapy or placebo procedure. Thus, we adopted TAC with a 25-guage needle instead of a 27-guage needle in the present study where LV pressure overload was relatively mild. Thus in the present study, the degree of LV dysfunction was mild and the beneficial effects of LIPUS on contractile function were also mild. And more, we have started the LIPUS therapy the day after TAC in the present study. Thus, it remains to be examined in future studies whether the LIPUS therapy ameliorates contractile dysfunction when started after development of HF. Third, in the chronic phase after TAC, mRNA expression of BNP was significantly lower in the TAC-LIPUS group than in the TAC-Control group, although there were no differences in heart weight, lung weight, or cross-sectional area of cardiomyocytes between the two groups. We consider that the difference in BNP reflects the degree of LV pressure overload and could be more evident in the more chronic phase. Further studies with severer HF model with a longer follow-up may be needed to address this point. Fourth, although we focused on angiogenesis and fibrosis as main mechanisms of the LIPUS-induced beneficial effects in the present study, other mechanisms of the LIPUS therapy remain to be examined, including those related to inflammation, reactive oxygen species, and wound healing [20,25,35]. Fifth, it remains to be examined whether the present protocol is optimal for the treatment of chronically pressure-overloaded hearts. Sixth, in the present study, the LIPUS therapy up-regulated protein levels of total eNOS, but not those of p-eNOS. Since it is reported that the eNOS expression in cardiac myocytes accounts for 20% of total cardiac eNOS [43], it is possible that the enhanced eNOS expression may represent an increased number of endothelial cells. This point remains to be examined in future studies. Further studies are needed to address this point.

## Conclusions

In the present study, we were able to demonstrate that the LIPUS therapy preserves contractile function in chronically pressure-overloaded hearts, associated with enhanced myocardial angiogenesis and attenuated perivascular fibrosis in mice in vivo. Thus, the LIPUS therapy may be a promising, novel, non-invasive therapy for cardiac dysfunction due to chronic LV pressure overload in humans.

## Supporting information

S1 Fig Study protocol (LIPUS during the first week alone). (A) Study protocol. LIPUS was applied to the whole heart only three times in the first week after TAC, while animals in the control group underwent the same procedures but without the LIPUS therapy. (B) Peak flow velocity at TAC. Results are expressed as mean±SD.
(TIF)

S2 Fig Echocardiographic data (LIPUS during the first week alone). (A) Representative echocardiographic images at 8 weeks after TAC. (B~G) Graphs showing the time course of anterior wall thickness (AWT) and posterior wall thickness (PWT) of the LV, LV dimension at end-diastole (LVDd), LVD at end-systole (LVDs), LV fractional shortening (LVFS), and LV ejection fraction (LVEF). Statistical analysis was performed at 8 weeks after TAC. Results are expressed as mean±SD.
(TIF)

S3 Fig Heart weight, lung weight and systolic blood pressure. (A) Heart weight/tibial length (HW/TL). (B) Lung weight/tibial length (LW/TL). (C) Systolic blood pressure during the therapy. Results are expressed as mean±SD.
(TIF)

S4 Fig The relationship between LVDd and LVDs. (A) Graphs showing the relationship between LVDd and LVDs in TAC-operated groups. (B) Graphs showing the relationship between LVDd and LVDs in Sham-operated groups.
(TIF)

S5 Fig Representative images of western blot in acute phase. These images showing p-eNOS, t-eNOS, p-Akt, t-Akt, VEGF and α-tubulin. Left; Control group. Right; LIPUS group.
(TIF)

S6 Fig Measurement of growth factors by Bio-Plex system in the acute phase. Graphs showing the levels of growth factors in the serum. bFGF, basic fibroblast growth factor; PDGF-BB, platelet-derived growth factor-BB; VEGF, vascular endothelial growth factor. Results are expressed as mean±SD.
(TIF)

S7 Fig Western blot analysis of bFGF and HGF in the acute and chronic phases. (A) Protein levels of HGF in the acute and chronic phases. (B) Protein levels of bFGF in the acute and chronic phases. Results are expressed as mean±SD.
(TIF)

S8 Fig Study protocol in Cav-1-KO mice. (A) Study protocol. LIPUS was applied to the whole heart three times in the first week after TAC and was thereafter repeated once a week for 7 weeks in the LIPUS group, while the control group underwent the same procedures but without the LIPUS therapy. (B) Study setup. (C) Peak flow velocity at TAC. (D) Heart weight/body weight (HW/BW). (E) Lung weight /body weight (LW/BW). Results are expressed as mean±SD.
(TIF)

S9 Fig Echocardiographic data in Cav-1-KO mice. (A) Representative echocardiographic images in Cav-1-KO mice at 8 weeks after TAC. (B~G) Graphs showing the time course of anterior wall thickness (AWT) and posterior wall thickness (PWT) of the LV, LV dimension at end-diastole (LVDd), LVD at end-systole (LVDs), LV fractional shortening (LVFS), and LV ejection fraction (LVEF). Results are expressed as mean±SD. Statistical analysis was performed at 8 weeks after TAC.
(TIF)

S1 Text Animal preparations in Cav-1-KO mice. (DOCX)
