# Supplementary figures and images for: Low-intensity pulsed ultrasound enhances angiogenesis and ameliorates contractile dysfunction of pressure-overloaded heart in mice
Source: PLoS One. 2017 Sep 28;12(9):e0185555. doi: 10.1371/journal.pone.0185555 (PMC5619801; doi:10.1371/journal.pone.0185555)

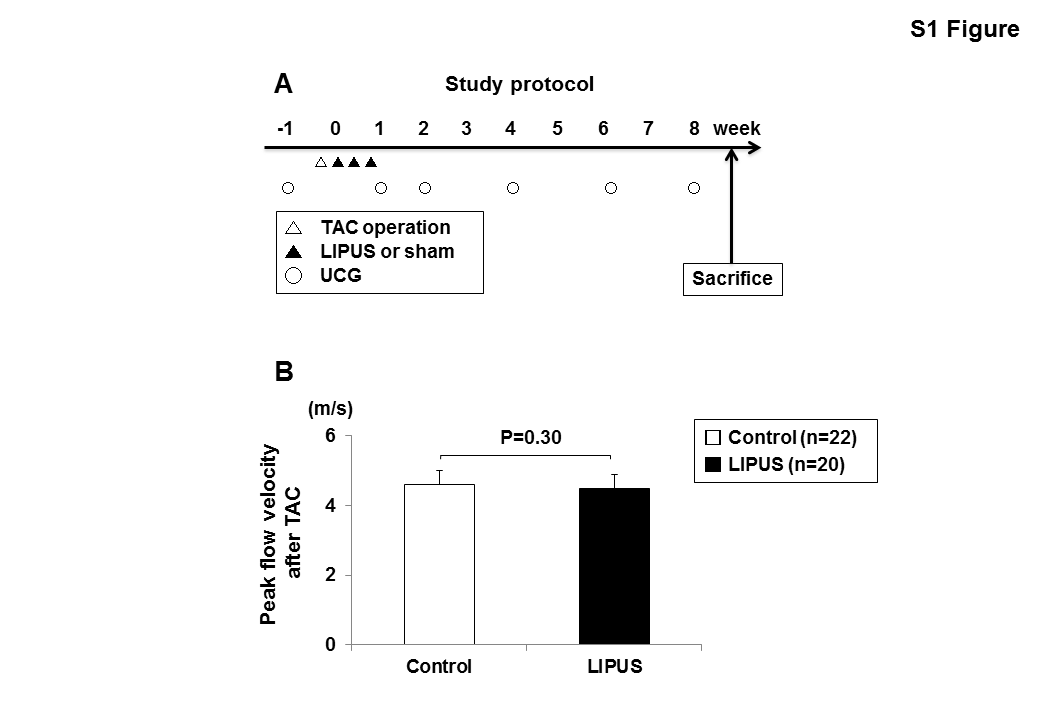

Supplement: S1 Fig — (A) Study protocol. LIPUS was applied to the whole heart only three times in the first week after TAC, while animals in the control group underwent the same procedures but without the LIPUS therapy. (B) Peak flow velocity at TAC. Results are expressed as mean±SD. (TIF) [file pone.0185555.s001.TIF]

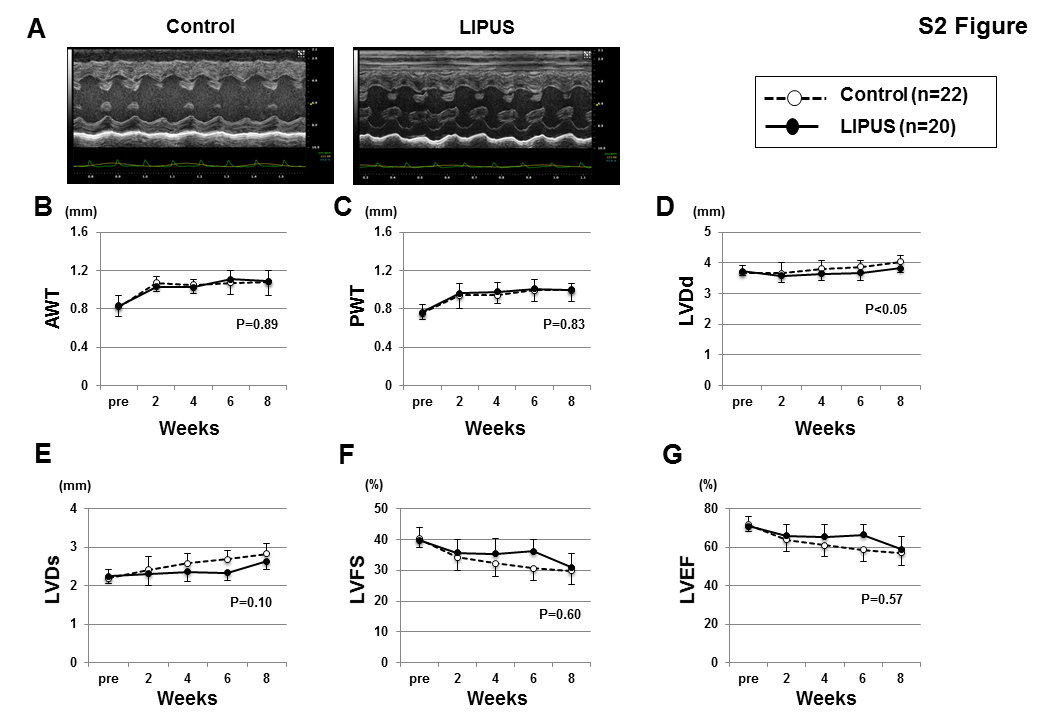

Supplement: S2 Fig — (A) Representative echocardiographic images at 8 weeks after TAC. (B~G) Graphs showing the time course of anterior wall thickness (AWT) and posterior wall thickness (PWT) of the LV, LV dimension at end-diastole (LVDd), LVD at end-systole (LVDs), LV fractional shortening (LVFS), and LV ejection fraction (LVEF). Statistical analysis was performed at 8 weeks after TAC. Results are expressed as mean±SD. (TIF) [file pone.0185555.s002.TIF]

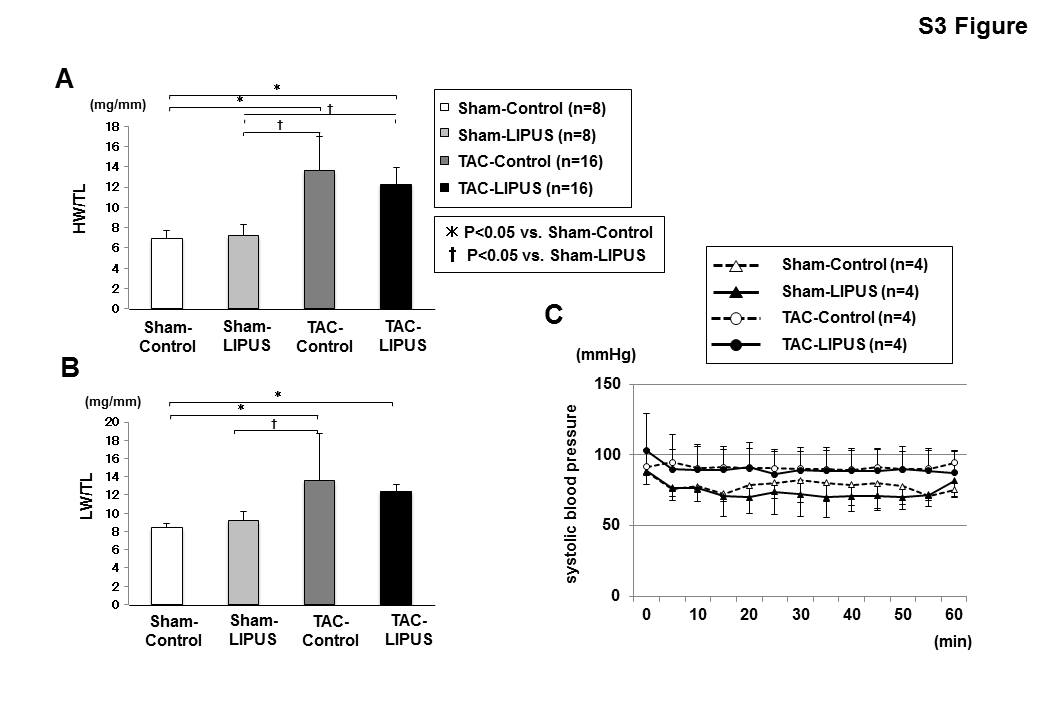

Supplement: S3 Fig — (A) Heart weight/tibial length (HW/TL). (B) Lung weight/tibial length (LW/TL). (C) Systolic blood pressure during the therapy. Results are expressed as mean±SD. (TIF) [file pone.0185555.s003.TIF]

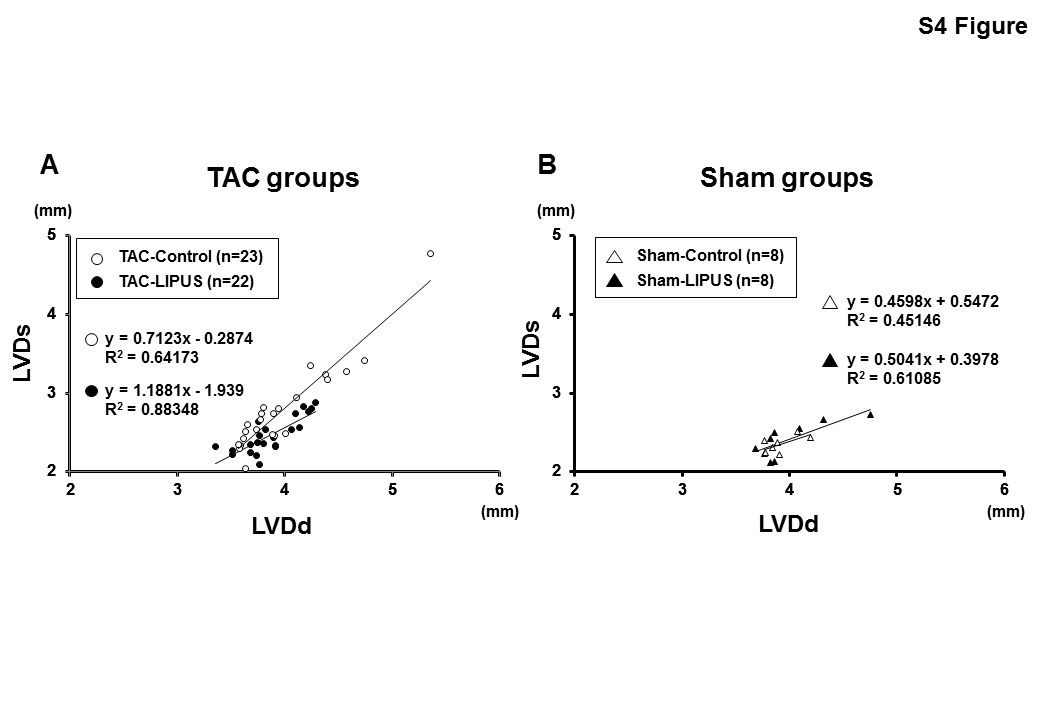

Supplement: S4 Fig — (A) Graphs showing the relationship between LVDd and LVDs in TAC-operated groups. (B) Graphs showing the relationship between LVDd and LVDs in Sham-operated groups. (TIF) [file pone.0185555.s004.TIF]

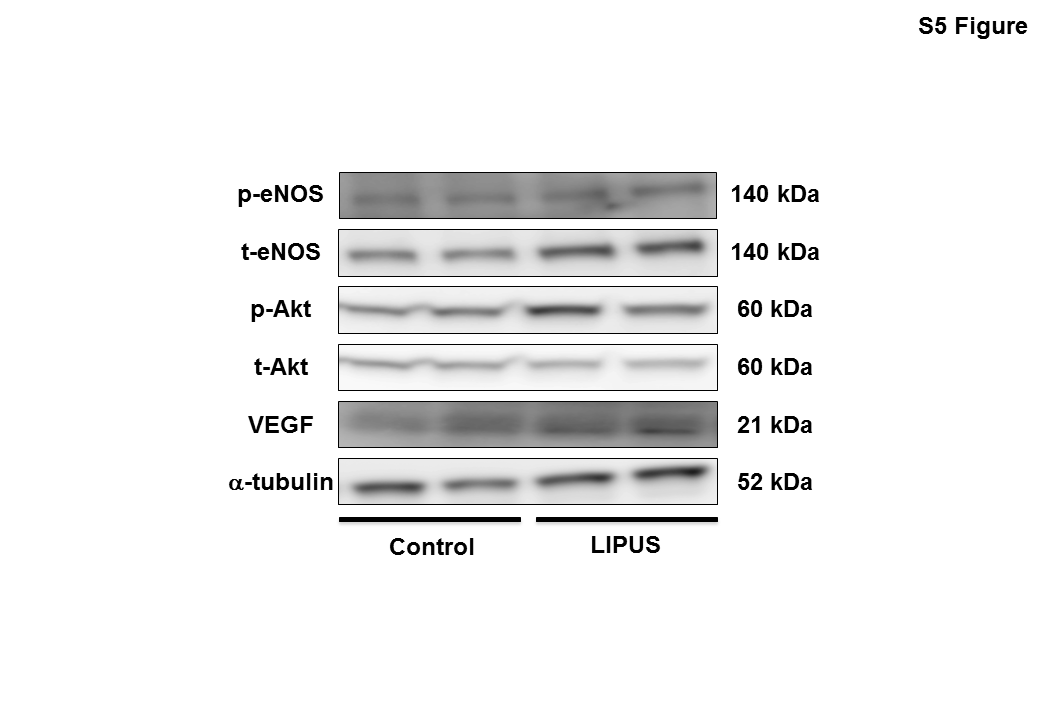

Supplement: S5 Fig — These images showing p-eNOS, t-eNOS, p-Akt, t-Akt, VEGF and α-tubulin. Left; Control group. Right; LIPUS group. (TIF) [file pone.0185555.s005.TIF]

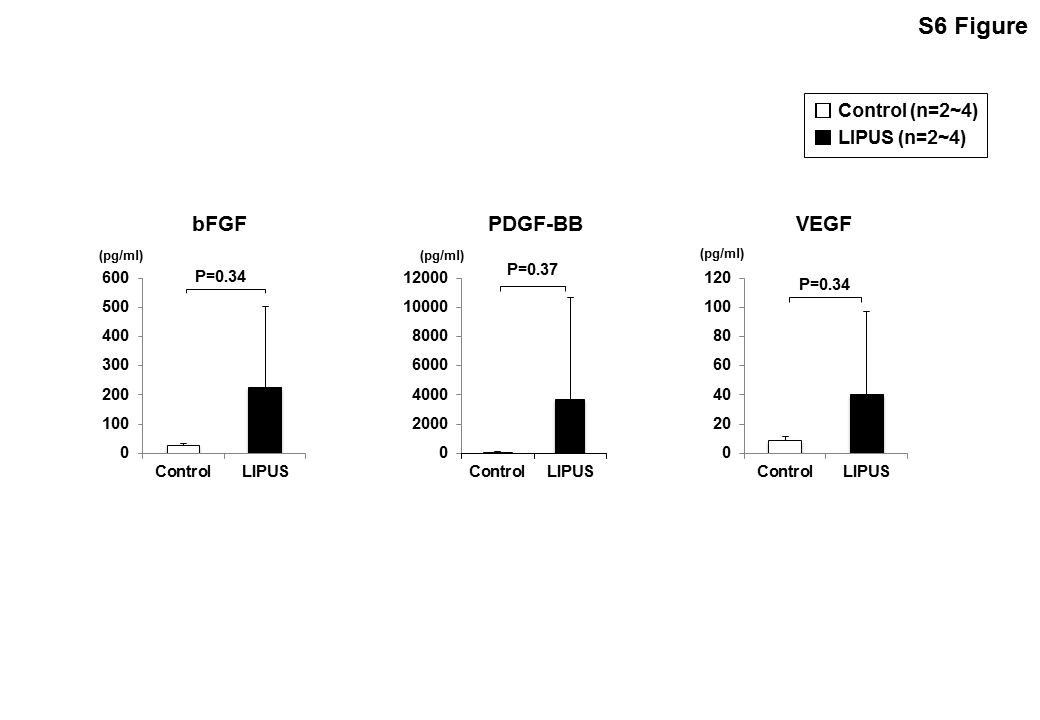

Supplement: S6 Fig — Graphs showing the levels of growth factors in the serum. bFGF, basic fibroblast growth factor; PDGF-BB, platelet-derived growth factor-BB; VEGF, vascular endothelial growth factor. Results are expressed as mean±SD. (TIF) [file pone.0185555.s006.TIF]

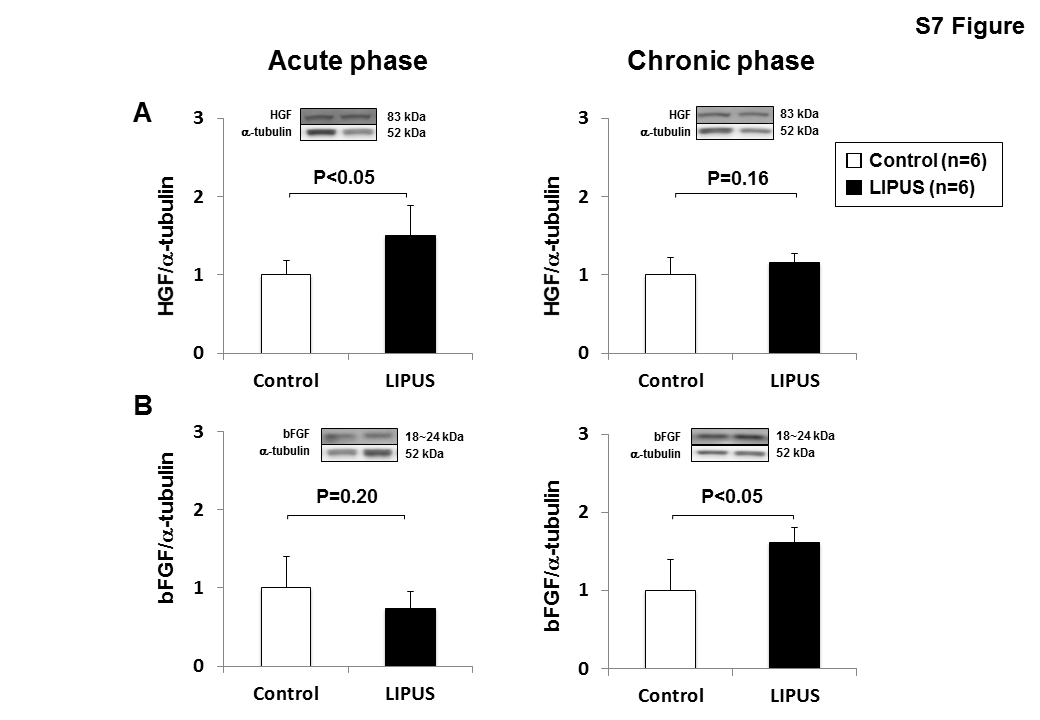

Supplement: S7 Fig — (A) Protein levels of HGF in the acute and chronic phases. (B) Protein levels of bFGF in the acute and chronic phases. Results are expressed as mean±SD. (TIF) [file pone.0185555.s007.TIF]

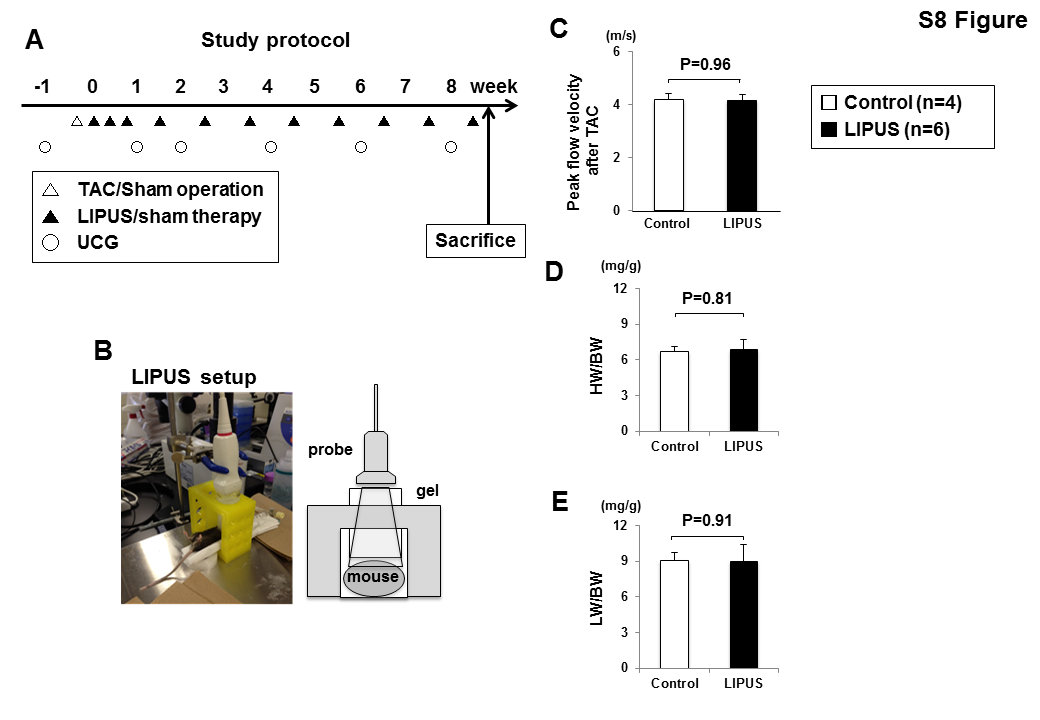

Supplement: S8 Fig — (A) Study protocol. LIPUS was applied to the whole heart three times in the first week after TAC and was thereafter repeated once a week for 7 weeks in the LIPUS group, while the control group underwent the same procedures but without the LIPUS therapy. (B) Study setup. (C) Peak flow velocity at TAC. (D) Heart weight/body weight (HW/BW). (E) Lung weight /body weight (LW/BW). Results are expressed as mean±SD. (TIF) [file pone.0185555.s008.TIF]

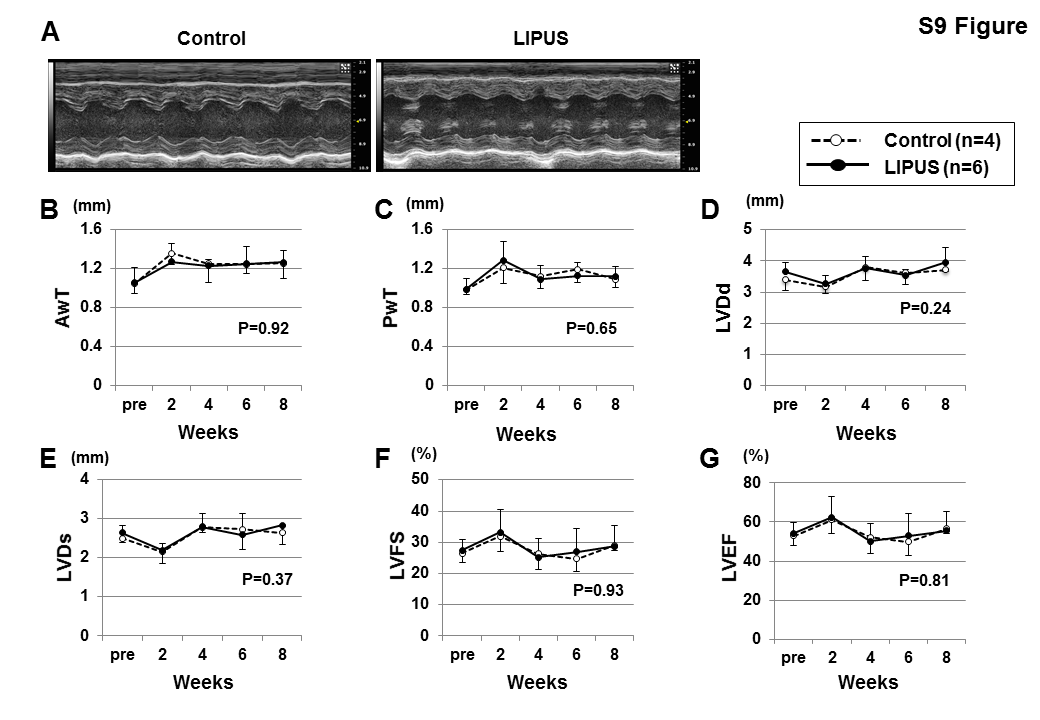

Supplement: S9 Fig — (A) Representative echocardiographic images in Cav-1-KO mice at 8 weeks after TAC. (B~G) Graphs showing the time course of anterior wall thickness (AWT) and posterior wall thickness (PWT) of the LV, LV dimension at end-diastole (LVDd), LVD at end-systole (LVDs), LV fractional shortening (LVFS), and LV ejection fraction (LVEF). Results are expressed as mean±SD. Statistical analysis was performed at 8 weeks after TAC. (TIF) [file pone.0185555.s009.TIF]
