# Supplementary material for: Low-intensity pulsed ultrasound enhances angiogenesis and ameliorates contractile dysfunction of pressure-overloaded heart in mice
Source: PLoS One. 2017 Sep 28;12(9):e0185555. doi: 10.1371/journal.pone.0185555 (PMC5619801; doi:10.1371/journal.pone.0185555)
Supplement: S1 Text — (DOCX) [file pone.0185555.s010.docx]

**Low-intensity Pulsed Ultrasound Enhances Angiogenesis and Ameliorates Contractile Dysfunction of Pressure-overloaded Heart in Mice**

Tsuyoshi Ogata^1^, Kenta Ito^1^*, Tomohiko Shindo^1^, Kazuaki Hatanaka^1^, Kumiko Eguchi^1^,

Ryo Kurosawa^1^, Yuta Kagaya^1^, Yuto Monma^1^, Sadamitsu Ichijo^1^, Hirofumi Taki^2,3^,

Hiroshi Kanai^2,3^, Hiroaki Shimokawa.^1^

^1^Department of Cardiovascular Medicine, Tohoku University Graduate School of Medicine, Sendai, Japan,

^2^Department of Electronic Engineering, Graduate School of Engineering, Tohoku University, Sendai, Japan,

^3^Division of Biomedical Measurements and Diagnostics, Graduate School of Biomedical Engineering, Tohoku University, Sendai, Japan.

**Short title**: LIPUS for Pressure-Overloaded Cardiac Dysfunction

**Key words**: angiogenesis, heart failure, cardiac function

*Address for Correspondence:

Kenta Ito, MD, PhD.

Department of Cardiovascular Medicine

Tohoku University Graduate School of Medicine

1-1 Seiryo-machi, Aoba-ku, Sendai 980-8574, Japan

TEL: +81-22-717-7153, FAX: +81-22-717-7156

E-mail: [ito-kenta@cardio.med.tohoku.ac.jp](mailto:ito-kenta@cardio.med.tohoku.ac.jp)

**Supporting Text 1.**

**Animal preparations**

All animal experiments were performed conform the NIH guidelines (Guide for the care and use of laboratory animals) and were conducted in accordance with the protocols approved by the Institutional Committee for Use and Care of Laboratory Animals of Tohoku University (2013 Idou-547). Caveolin-1-deficient (Cav-1-KO) mice (15-22-week-old, 23-30 g in body weight) also underwent transverse aortic constriction (TAC) to induce chronic LV pressure overload. Cav-1-KO mice were developed by breeding pairs of heterozygous mice (Jackson Laboratory, Barharbor, ME, USA) and maintained in our institute [27]. Cav-1 KO mice are smaller than wild-type mice at the same age. To perform TAC surgery, we used older Cav-1-KO mice than C57/Bl6 wild type mice to adjust body weight in order to create the same extent of pressure overload to the heart.
